# Stroke in Toddler After Minor Head Injury: An Emerging Diagnostic Challenge

**DOI:** 10.7759/cureus.35479

**Published:** 2023-02-26

**Authors:** Kazuyuki Miyamoto, Hiromi Takayasu, Atsuo Maeda, Jun Sasaki, Munetaka Hayashi

**Affiliations:** 1 Department of Emergency Medicine, Showa University Northern Yokohama Hospital, Yokohma, JPN; 2 Department of Emergency, Critical Care Medicine, Showa University Fujigaoka Hospital, Yokohama, JPN; 3 Department of Emergency Care Medicine, Showa University Koto Toyosu Hospital, Tokyo, JPN

**Keywords:** delayed diagnosis, magnetic resonance imaging, emergency physician, minor head injury, toddler stroke

## Abstract

The diagnosis of ischemic stroke in toddlers in ED is a challenge due to non-specific neurological symptoms and difficulties in conducting a detailed neurological examination in toddlers. Magnetic Resonance Imaging (MRI) requires patient sedation and the cooperation of several medical personnel. A 33-month-old male presented with the immobility of the left upper extremity after a fall from a child chair. A head computerized tomography scan revealed no obvious bleeding. An orthopedic surgeon, a neurosurgeon, and a pediatrician were consulted but could not provide a definitive diagnosis. The following day, the patient developed left incomplete hemiplegia and dysarthria, and an emergency MRI detected a high signal at the right nucleus basalis. The patient was diagnosed with acute cerebral infarction and transferred to a children’s hospital. Pediatric minor head injuries and pulled elbows are commonly presented in ED, and most patients are discharged safely. Despite persistent neurological deficits several hours after arrival, we could not perform an MRI, which delayed the diagnosis. We recommend that early MRIs are performed in similar cases to aid rapid diagnoses. The collaboration between several specializations allowed the successful diagnosis and treatment of this case.

## Introduction

The diagnosis of ischemic stroke among toddlers in ED is challenging. The incidence of cerebral infarction in children is rare, and the annual incidence is 1.72 per 100,000 people a year [1]. Emergency physicians have few opportunities to diagnose strokes in toddlers in the emergency department (ED); they may not present with specific focal neurologic symptoms, and a detailed neurological examination is difficult to carry out, which can result in misdiagnosis or a delayed diagnosis [2,3]. The mean time from symptoms onset to disease diagnosis is 22.7 h [4], stroke-specific mortality is 5%, and neurological deficits are 70% in children [1]. Urgent evaluation and neuroimaging are vital for the early diagnosis of strokes in toddlers for better outcomes. Brain Magnetic Resonance Imaging (MRI) and Magnetic Resonance Angiography (MRA) are suitable for evaluating acute ischemia. However, unlike adults, sedation or general anesthesia is usually required to minimize motion artifacts while conducting an MRI on a toddler [5]. MRI scanning in toddlers requires the cooperation of an anesthesiologist, pediatrician, and radiology technician.

## Case presentation

A 33-month-old male presented to ED with a complaint of immobility of the left upper extremity. He was otherwise healthy. On the morning of the accident, he behaved as usual and changed his clothes by himself after waking up. While eating breakfast, he fell from a child's chair approximately 50 cm in height. The fall was not witnessed, but the child was found on the floor by the mother. The patient continued eating breakfast using both hands after the accident but gradually lost the ability to grasp objects with the left upper limb. The patient had a history of four incidents of a pulled right elbow but was not taking any medications. On arrival at the hospital, the patient was irritated and crying. There was no obvious head trauma, such as subcapsular hematoma or contusion. As the patient was noncooperative, a detailed neurological examination could not be conducted.

**Figure 1:**
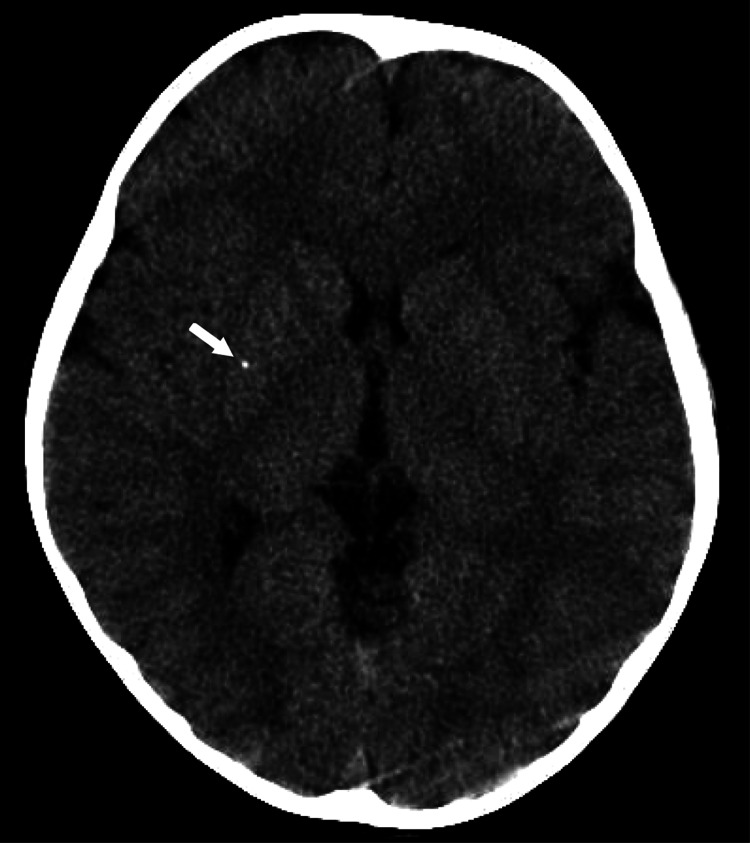
Head computed tomography (CT) scan on day one. There was a motion effect associated with body movement, though there was no obvious bleeding or fractures. The head CT taken on the first day was later reviewed to find mineralizing angiopathy (arrow) at the right basal ganglia.

We detected a motion effect associated with body movement on the head by computed tomography (CT), although there was no obvious bleeding or fractures (Figure 1). No fracture was detected on an X-ray of the left elbow. Due to the difficulty in diagnosis, an orthopedic surgeon, a neurosurgeon, and a pediatrician were consulted. The orthopedic surgeon reported no tenderness or swelling of the left elbow. The patient was subjected to the reduction technique but didn't feel any apparent clicks. The orthopedic surgeon provided a possible reason for the patient’s inability to move the left extremity was due to pain after presumed post-reduction. The neurosurgeon commented that the traumatic head injury was unlikely to be related to the patient’s symptoms. The pediatrician’s examination, including the laboratory tests, could not detect the cause of the symptoms. At this point, six hours had passed since the patient’s arrival, and he was therefore admitted for observation. The patient fell asleep shortly after admission. On the second morning, the patient developed drooling from the left corner of the mouth, left incomplete hemiplegia, and dysarthria.

**Figure 2:**
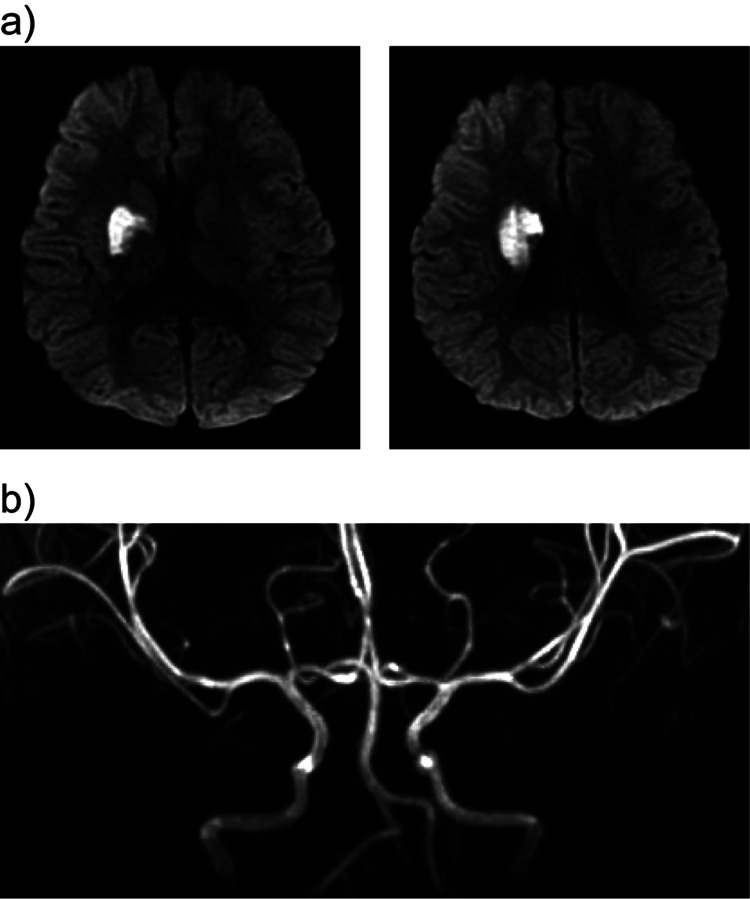
Head Magnetic Resonance Imaging (MRI) with diffusion-weighted imaging (DWI) and Magnetic Resonance Angiography (MRA) on day two. a) A high signal was detected at the right nucleus basalis in DWI. b) An MRA showed no obvious abnormalities.

Head MRI with diffusion-weighted imaging (DWI) performed under sedation detected a high signal at the right nucleus basalis (Figure 2a), but MRA showed no obvious abnormalities (Figure 2b). The definitive diagnosis was acute cerebral infarction in the perforator of the middle cerebral artery. Aspirin was administered, and the patient was transferred to a children's hospital. A detailed examination of thrombophilia and echocardiography revealed no abnormality, and he was diagnosed with infarction after minor head injury. The patient was fortunately discharged without any neurological sequelae after rehabilitation.

## Discussion

The etiologies and risk factors for ischemic stroke in children are cardiac abnormalities, vascular lesions, hematologic abnormalities, and head trauma [6]. Children with minor head injury (MHI) rarely have a basal ganglionic infarction. Shearing injury due to angular acceleration forces after MHI can cause the occlusion or spasm of the perforator of the nucleus basalis [5,7]. A similar study reported post-traumatic infarction with speech disturbance two hours after MHI in a 4-year-old patient [8]. In our patient, paralysis of the left upper extremity appeared a while after MHI. Hence, it was thought that the patient had a stroke after MHI rather than an MHI due to a stroke. The mineralization of the lenticulostriate arteries was associated with infantile basal ganglia stroke after MHI [9]. We reviewed the head CT taken on the first day and identified mineralizing angiopathy at the right basal ganglia, which was a risk factor for stroke (Figure 1). MHI and pulled elbow in children are commonly presented in ED, and most patients are discharged safely [10]. Several physicians, including us, examined the patient individually from professional perspectives, but the patient’s history of the pulled elbow and MHI made the disease diagnosis ambiguous. Despite persistent neurological deficits several hours after arrival, we could not perform MRI, which delayed the diagnosis. Fortunately, the patient’s neurological symptoms were not severe and did not progress due to the delay in performing an MRI and making a definitive diagnosis. However, it is important to conduct an emergency MRI in cases with similar neurological symptoms to facilitate rapid diagnosis and early treatment. Considering the patient’s risk factors and symptoms, the emergency physician should have taken the initiative in making the definitive diagnosis at ED.

## Conclusions

MRI scanning in children requires sedation or general anesthesia and cooperation between medical personnel of several departments. Hence, emergency physicians should take the initiative and collaborate with other specialist physicians to make the definitive diagnosis.
